# Supplementary material for: Detection of stable community structures within gut microbiota co-occurrence networks from different human populations
Source: PeerJ. 2018 Feb 7;6:e4303. doi: 10.7717/peerj.4303 (PMC5807925; doi:10.7717/peerj.4303)
Supplement: Supplemental Information 1 — An overview of code and scripts used in main stages of network creation and community detection. [file peerj-06-4303-s001.pdf]

Outline of main scripts used to generate community definitions.

This supplement contains details of code used for generation of OTUs, co-occurrence networks and community definitions. Unless highlighted otherwise, the code refers to shell commands referencing existing software. Additional reformatting of data was carried out using both custom scripts and manual curation, details of which can be found in methods or on request.

Generation of OTUs from combined dataset

16S sequencing data from all three data sets was combined with read headers having the format SampleID_ReadNumber. OTU creation was carried out using vsearch 2.4 as below.

Dereplicate sequences
```
vsearch_2.4.0/bin/vsearch --derep_full complete_twins_israeli_lldeep.fna --
output all_derep.fna --log=log --sizeout --minuniquesize 2
```
Find centroids (representative sequences)
```
vsearch_2.4.0/bin/vsearch -cluster_fast all_derep.fna -id 0.97 --sizein --
sizeout --relabel OTU_  --centroids all_otus.fna
```
Remove chimeric representative sequences
```
vsearch_2.4.0/bin/vsearch --uchime_denovo all_otus.fna --nonchimeras
otus_checked.fna --sizein --xsize --chimeras chimeras.fasta
```
Cluster reads against representative sequences
```
vsearch_2.4.0/bin/vsearch -usearch_global complete_twins_israeli_lldeep.fna
-db otus_checked.fna -strand plus -id 0.97 -uc otu_table_mapping.uc
```
Generate QIIME compatible biom table from uc OTU file
```
biom from-uc -i otu_table_mapping.uc -o vsearch_otus.biom
```

Splitting, summarising and sub-setting of OTU tables was carried out using QIIME, as was taxonomic assignment of OTUs, phylogenetic tree generation and beta and alpha diversity analyses (detailed in methods).

Calculation of co-occurrence between OTUs

OTU tables were split by dataset, then each dataset's table filtered to only include OTUs found in at least 25% of its samples. Four co-occurrence measures were then calculated from tab-delimited text tables for each data set as below (shown are examples for TwinsUK but this was carried out on all three datasets). All co-occurrence measures were calculated on a high performance compute cluster, in parallel where possible.

SparCC

Calculate SparCC correlation between real OTU counts
```
python SparCC.py TwinsUK_25per_raw_counts.txt --
cor_file=TwinsUK_SparCC_Cor.txt
```
Generate 100 bootstrapped OTU count tables
```
python MakeBootstraps.py TwinsUK_25per_raw_counts.txt -n 100 -t
permutation_#.txt -p TwinsUK_Boots
```
Calculate SparCC correlation on each of the bootstrapped tables (# = 1-100)
```
python SparCC.py TwinsUK_Boots/TwinsUK_Bootspermutation_#.txt --cor_file=
TwinsUK_BootCors/TwinsUK_BootsCor_#.txt
```
Combine bootstrapped and real correlation values to calculate p-values for correlations

```
python PseudoPvals.py TwinsUK_SparCC_Cor.txt TwinsUK_BootsCor_#.txt 100 -o
TwinsUK_SparCC_Pvals_TwoSided.txt -t two_sided
```

CoNet

Script to run correlation analysis between OTUs on a sun grid engine managed compute cluster using command line CoNet. Generates a CoNet .gdl network file that is manually converted to an edge table using Cytoscape. Takes the >25 percent of samples OTU table converted to relative abundances as input.

```
#Settings to direct to local Java and CoNet installs
JAVA=java
MEM=17000
LIB=CoNet/lib/CoNet.jar
#Locations of inputs and outputs
INPUTFOLDER=Input
OUTPUTFOLDER=Output
#file name of input
MATRIX=TwinsUK_25per_relative_abund.txt
RESULT_NAME=TwinsUK_CoNet
#CoNet parameters
METHODS=correl_spearman/correl_pearson/dist_kullbackleibler/dist_bray/
EDGE_NUMBER=2000
ITERATIONS=1000
NETWORK_MERGE=union
RENORM="--renorm"
RANDROUTINE=edgeScores
MULTITESTCORR=benjaminihochberg
RESAMPLING=shuffle_rows
PVAL_MERGE=simes
PVAL_THRESHOLD=0.05
NULLDISTRIBS=$RESULT_NAME"_permutations.txt"
#specify output to GDL network
FORMAT=gdl
#configuration files that specify number of jobs to run on cluster etc.
examples can be found in CoNet documentation
CONFIG=CoNetConfig.txt
CONFIGBOOT=CoNetConfigBoot.txt
CLUSTER="-b"

#find initial thresholds based on number of edges
$JAVA -Xmx"$MEM"m -cp "$LIB"
be.ac.vub.bsb.cooccurrence.cmd.CooccurrenceAnalyser -i $INPUTFOLDER/$MATRIX
-E $METHODS --thresholdguessing edgeNumber --guessingparam $EDGE_NUMBER --
method ensemble -o $OUTPUTFOLDER/"$RESULT_NAME"_thresholds.txt --topbottom
-Z $INPUTFOLDER/$CONFIG > $OUTPUTFOLDER/"$RESULT_NAME".log

#permute for reboot handling of compositionality
$JAVA -Xmx"$MEM"m -cp "$LIB"
be.ac.vub.bsb.cooccurrence.cmd.CooccurrenceAnalyser -i $INPUTFOLDER/$MATRIX
-E $METHODS --method ensemble -f $FORMAT -o
$OUTPUTFOLDER/"$RESULT_NAME"_ensemble.gdl --ensembleparamfile
$OUTPUTFOLDER/"$RESULT_NAME"_thresholds.txt --networkmergestrategy
$NETWORK_MERGE --multigraph --pvaluemerge $PVAL_MERGE -F rand $RENORM --
iterations $ITERATIONS -g $PVAL_THRESHOLD --resamplemethod $RESAMPLING -I
$OUTPUTFOLDER/$NULLDISTRIBS -K $RANDROUTINE --scoreexport $CLUSTER -Z
$INPUTFOLDER/$CONFIG >> $OUTPUTFOLDER/"$RESULT_NAME".log
```

```
#bootstrap for reboot handling of compositionality
RESAMPLING=bootstrap
$JAVA -Xmx"$MEM"m -cp "$LIB"
be.ac.vub.bsb.cooccurrence.cmd.CooccurrenceAnalyser -i $INPUTFOLDER/$MATRIX
-E $METHODS --method ensemble -f $FORMAT -o
$OUTPUTFOLDER/"$RESULT_NAME"_ensemble.gdl --ensembleparamfile
$OUTPUTFOLDER/"$RESULT_NAME"_thresholds.txt --networkmergestrategy
$NETWORK_MERGE --multigraph --pvaluemerge $PVAL_MERGE -F rand --iterations
$ITERATIONS -g $PVAL_THRESHOLD --resamplemethod $RESAMPLING -I
$OUTPUTFOLDER/"$RESULT_NAME"_bootstraps.txt -K $RANDROUTINE --scoreexport
$CLUSTER -Z $INPUTFOLDER/$CONFIGBOOT >> $OUTPUTFOLDER/"$RESULT_NAME".log

#combine previous steps and generate GDL network file
$JAVA -Xmx"$MEM"m -cp "$LIB"
be.ac.vub.bsb.cooccurrence.cmd.CooccurrenceAnalyser -i $INPUTFOLDER/$MATRIX
-E $METHODS --method ensemble -f $FORMAT -o
$OUTPUTFOLDER/"$RESULT_NAME"_ensemble.gdl --ensembleparamfile
$OUTPUTFOLDER/"$RESULT_NAME"_thresholds.txt --networkmergestrategy
$NETWORK_MERGE --multigraph --pvaluemerge $PVAL_MERGE -F
rand/confidence_boot --iterations $ITERATIONS -g $PVAL_THRESHOLD --
resamplemethod $RESAMPLING -I $OUTPUTFOLDER/"$RESULT_NAME"_bootstraps.txt -
K $RANDROUTINE --multicorr $MULTITESTCORR --nulldistribfile
$OUTPUTFOLDER/$NULLDISTRIBS -Z $INPUTFOLDER/$CONFIG >
$OUTPUTFOLDER/"$RESULT_NAME"_restore.log
```

Pearson's and Spearman's Co-efficients

Pearson's and Spearman's co-efficients were calculated from the relative abundance tables (as used for CoNet) using the following R script.

```
#Read in the abundance table
abundtab=read.table(TwinsUK_25per_relative_abund.txt,header=T,sep='\t',row.
names=1)

#Hmisc library used to calculate coefficients and p-values
library(Hmisc)

#Calculate Pearson coefficients and p-values, write coefficients
Pears=rcorr(t(as.matrix(abundtab)),type="pearson")
write.table(Pears$r,'TwinsUK_pearson_corr_coefss.txt',sep='\t',col.names=NA
)

#work out how many unique correlations calculated, for use in FDR
correction
n=nrow(abundtab)
gridsize=n*n
digrem=gridsize-n
numuniquecomp=digrem/2

#correct the pvalues for the Pearson correlation using Bonferroni and
number of tests as above
ltri=lower.tri(Pears$P)
pmat=Pears$P
pmat[ltri]=p.adjust(pmat[ltri],method="bonferroni",n=numuniquecomp)
write.table(pmat,TwinsUK_pearson_corr_bonf_ps_on_lower.txt',sep="\t",col.na
mes=NA)

#Repeat above but for Spearman
Spear=rcorr(t(as.matrix(abundtab)),type="spearman")
```

```
write.table(Spear$r,’TwinsUK_spearman_corr_coefss.txt',sep='\t',col.names=N
A)

ltri2=lower.tri(Spear$P)
pmat2=Spear$P
pmat2[ltri]=p.adjust(pmat2[ltri2],method="bonferroni",n=numuniquecomp)
write.table(pmat2,’TwinsUK_spearman_corr_bonf_ps_on_lower.txt',sep="\t",col
.names=NA)
```

The edge tables for all these approaches was converted to an unweighted edge table format as below:

OTU1	OTU2	Direction(1 or -1)	Corrected P-value
Denovo1	Denovo2	1	0.001

Generating intersect networks

The edge tables above were thresholded at different p-values (removing rows who’s p-values were above the threshold). This was done at a range of p-values, for every co-occurrence measure and for each data set. The resultant edge tables were combined across the four co-occurrence measures at each p-value generate intersect networks using the following python script (which was run at each p-value for each data set).

```
“””takes input edge files for 4 co-occurrence measures at a pre-thresholded
p-value and generates intersect network (retains edges in the same
direction in all 4)”””

import sys, re
#create output file and write a header
outfile=open(“TwinsUK_Intersect_p0_01.txt”,'w')
outfile.write("Node1\tNode2\tDirection\n")

#list of co-occurrence measures
networks=[]

#go through the edge tables provided as a list in command line
for i in range(len(sys.argv[1:])):
	#make dictionary to store the table in for each method
	networks.append({})
	fileopen=open(sys.argv[i+2],'rU').readlines()
	for j in range(len(fileopen)):
		if j == 0:
			pass
		else:
			row=fileopen[j].strip('\n').split('\t')
			corr=float(row[2])
			direct=2
			if corr > 0:
				direct=1
			elif corr < 0:
				direct=-1
			#add edge to dictionary for both possible orientations
			networks[i][(node1,node2)]=direct
			networks[i][(node2,node1)]=direct

#go through first network and find matches in other two and write those out
#skip ones where other orientation already done
skiplist=[]
for i in networks[0]:
```

```
        if i in skiplist:
                pass
        else:
                node1=i[0]
                node2=i[1]
                firstdir=networks[0][i]
                skiplist.append((node2,node1))
                #check in other networks for matches
                keep=True
                for j in networks[1:]:
                        if keep == True:
                                if i not in j:
                                        keep=False
                                elif j[i] != firstdir:
                                        keep=False
                if keep == True:
                        outfile.write(node1+"\t"+node2+"\t"+str(firstdir)+"\n")

outfile.close()
```

Measuring degree distribution and calculating power law fit
The resultant intersect edge tables at each p-value were assessed to calculate their degree distribution using the following python script (for each data set independently).

```
“””script run as python degree_dist.py TwinsUK_node_list.txt
TwinsUK_Degree_Dist_Output.txt
followed by a list of the interesct network edge tables at all the p-
values”””

import sys
import networkx as nx

#create output file
outfile=open(sys.argv[2],'w')

#dictionary to store degree distribution at each p-value
degreedict={}

#add nodes not in the edge table (no connections)
nodes=open(sys.argv[1],'rU').readlines()
nodelist=[]
for i in nodes:
        nodelist.append(i.strip("\n"))


#for each p-value intersect file given produce the degree distribution
for i in sys.argv[3:]:
        fileopen=open(i,'rU').readlines()
        degreedict[i]=[]
        #create a graph for the network
        g=nx.Graph()
        #found nodes
        foundnodes=[]
        for j in range(len(fileopen)):
                if j==0:
                        #skip header
                        pass
                else:
```

```
                  row=fileopen[j].strip('\n').split("\t")
                  g.add_edge(row[0],row[1],weight=int(row[2]))
                  foundnodes.append(row[0])
                  foundnodes.append(row[1])
      for j in nodelist:
            if j in foundnodes:
                  pass
            else:
                  g.add_node(j)
      for node in g.nodes():
            degreedict[i].append(g.degree(node))


longest=0
for i in degreedict:
      if len(degreedict[i]) > longest:
            longest=len(degreedict[i])

for i in degreedict:
      row=i.strip(".txt").split("_")[-1]
      for j in range(longest):
            try:
                  row+="\t"+str(degreedict[i][j])
            except:
                  row+="\tNA"
      row+="\n"
      outfile.write(row)

#output will contain distribution at each p-value for power fit calculation
in R
outfile.close()
```

The fit of the distributions for each p-value intersect was calculated suing the following R script.

```
#load WGCNA
library(WGCNA)

#load degree distributions from previous python script across all p-values
degrees=read.table(TwinsUK_Degree_Dist_Output.txt,header=F,sep="\t")

#store results for each p-value
pvals=c()
meandeg=c()
totedges=c()
R2=c()
slope=c()

#at each p-value threshold used to generate intersect networks find the
networks fit to a #power law distribution using the WGCNA function
extracting slope and R2
for(i in 1:nrow(degrees)){
  pval=degrees[i,1]
  pvals=c(pvals,pval)
  deg=degrees[i,2:ncol(degrees)]
  deg=na.omit(as.numeric(deg))
  meandeg=c(meandeg,mean(deg))
  totedges=c(totedges,(sum(deg))/2)
  ft=scaleFreeFitIndex(deg, nBreaks = 10, removeFirst = FALSE)
  R2=c(R2,ft$Rsquared.SFT)
```

```
  slope=c(slope,ft$slope.SFT)
}

res=data.frame(p=pvals,r2=R2,meand=meandeg,toted=totedges,slop=slope)

write.csv(res,args[2])
```

Finding the γ value to use in genLouvain modularity maximisation

After selecting an appropriate p-value threshold that generated an intersect network fitting a power law degree distribution in all three networks ($p<0.01$), we then aimed to find the optimum γ value to use in the genLouvain algorithm to identify communities within these networks. This was achieved by applying the following MATLAB script to the three data sets. This carried out community detection at a range of γ values, 25 times on the real network and on 100 randomised networks. It outputs the modularity observed in the real and random runs and the normalised variation of information calculated pairwise between all 25 real runs. Input was an adjacency matrix created from the intersect edge table ignoring negative edges. This is largely based on example scripts accompanying the genLouvain documentation.

```
%%
%add the script directories
addpath('./GenLouvain2.0')
addpath('./octave_networks')

%%
%import the adjacency matrix
adjmat=readtable(TwinsUK_AdjMat_p0_01.txt,
'Delimiter','\t','ReadVariableNames',true,'ReadRowNames',true);
A=table2array(adjmat);

%set number of random networks to use to generate the null modularity
distribution
numrands = 100;
%using the octave library get the degree sequence from the adjacency matrix
[totdeg,indeg,outdeg]=degrees(A);
%sort the total degree large to small
degseq=sort(totdeg,'descend');

%%
%set the gamma range and number of iterations

%gamma value range to consider
gamma = [0.01:0.01:3];
its = 25;

%%
%generate the node edge summaries
k = full(sum(A));
twom = sum(k);

%%
%lists to store means
ModMeans=[];
StabMeans=[];
NumberClusMeans=[];
NumberBigClusMeans=[];
NullModMeans=[];
```

```
%%
%lists to store SDs
ModSDs=[];
StabSDs=[];
NumberClusSDs=[];
NumberBigClusSDs=[];
NullModSDs=[];

%matrix to store max mod partition at each res
maxparts=zeros(size(A,1),length(gamma));

%%
%for each resolution do resolutions and write out the data and find the
%best partition
for i=1:length(gamma)
    disp(['Gamma Resolution:' num2str(gamma(i)) '   ' num2str(i) '/'
num2str(length(gamma))])
    %%
    %store modularities for each run
    res_mods=[];
    %%store partitions for each run
    parts=zeros(size(A,1),its);
    %%store number of clusters
    num_clus=[];
    %%store number of clusters > 3 nodes
    num_clus_big=[];

    %%
    %store max mod at each resolution
    maxmod=0;
    %for number of iterations
    for j=1:its
        %%
        %carry out modularity maximisation
        B = full(A - gamma(i)*k'*k/twom);
        [S,Q] = genlouvain(B,10000,0);
        %normalise modularity
        Q = Q/twom;
        %add modularity to iteration vector
        res_mods=[res_mods Q];
        %add partition to iteration matrix (for stability later)
        parts(:,j)=S;
        %if modularity higher than any other in this res add to max matrix
        if Q > maxmod
            maxparts(:,i)=S;
            maxmod=Q;
        end
        %find number of clusters and add to iteration vector
        uniqueclus=unique(S);
        cluscount=length(uniqueclus);
        num_clus=[num_clus cluscount];

        %%
        %find number of clusters >3 and add to iteraction vectors
        n=0;
        for h=1:cluscount
          if sum(S==uniqueclus(h))>2
              n=n+1;
          end
        end
```

```
        num_clus_big=[num_clus_big n];
        %
    end

    %%
    %add the means and sds from the iterations to the resolution comparison
    %vectors
    ModMeans=[ModMeans mean(res_mods)];
    NumberClusMeans=[NumberClusMeans mean(num_clus)];
    NumberBigClusMeans=[NumberBigClusMeans mean(num_clus_big)];
    ModSDs=[ModSDs std(res_mods)];
    NumberClusSDs=[NumberClusSDs std(num_clus)];
    NumberBigClusSDs=[NumberBigClusSDs std(num_clus_big)];

    %%calculate a null mod dist at this gamma

    %store modularities for each random graph
    randmods=[];
    %generate random graphs from degree seq and calc mod at the gamma for
each
    parfor y=1:numrands
         randA=randomGraphFromDegreeSequence(degseq);
         %generate the node edge summaries
         kR = full(sum(randA));
         twomR = sum(kR);
         %carry out modularity maximisation
         BR = full(randA - gamma(i)*kR'*kR/twomR);
         [SR,QR] = genlouvain(BR,10000,0);
         %normalise modularity
        QR = QR/twomR;
        randmods=[randmods QR];
    end
    NullModMeans=[NullModMeans mean(randmods)];
    NullModSDs=[NullModSDs std(randmods)];

    %%
    %calculate pairwise distances between all the iteration partitions (in
    %parallel)
    disp('Calculating pairwise Variation of Information...')
    distances=[];
    parfor l=1:its
        for m=l+1:its
           [zR,sR,sAR,vdist]=zrand(parts(:,l),parts(:,m));
           %normalise from 0-1
           vdist=vdist/log(length(parts(:,l)));
           distances=[distances vdist];
        end
    end
    disp(length(distances));
    %add the means and sd
    StabMeans=[StabMeans mean(distances)];
    StabSDs=[StabSDs std(distances)];


end
%

%%
%store results in a table
```

```
res=table(gamma',ModMeans',ModSDs',StabMeans',StabSDs',NumberClusMeans',Num
berClusSDs',NumberBigClusMeans',NumberBigClusSDs',NullModMeans',NullModSDs'
);
res.Properties.VariableNames = {'Gamma_Resolution' 'Mean_Modularity'
'SD_Modularity' 'Mean_Variation_of_Information'
'SD_Variation_of_Information' 'Mean_Number_of_Clusters'
'SD_Number_of_Clusters' 'Mean_Number_of_Clusters_Over_2_Nodes'
'SD_Number_of_Clusters_OVer_2_Nodes','Null_Modularity_Mean','Null_Modularit
y_SD'};
%write to a file
writetable(res,'TwinsUK_gamma_scan_results.txt');
exit;
```

Generating final community definitions

After selecting the final γ gamma value to use to define communities (0.4) we ran the community detection 100 times on each data set and retained the community definitions with the highest modularity value. This was achieved using a MATLAB script adapted from the previous one, as below.

```
%%
%add the script directories
addpath('GenLouvain2.0')
addpath('./octave_networks')

%%
%import the adjacency matrix d
adjmat=readtable(TwinsUK_AdjMat_p0_01.txt,
'Delimiter','\t','ReadVariableNames',true,'ReadRowNames',true);
A=table2array(adjmat);

%%
%set the gamma and number of iterations
gamma =str2num(gammavalue);
its = 100;

%%
%generate the node edge summaries
k = full(sum(A));
twom = sum(k);

disp(gamma);

%store max mod at each resolution
maxmod=0;
%for number of iterations
for j=1:its
    %%
    %carry out modularity maximisation
    B = full(A - gamma*k'*k/twom);
    [S,Q] = genlouvain(B,10000,0);
    %normalise modularity
    Q = Q/twom;
    %if modularity higher than any other in this res add to max matrix
    if Q > maxmod
        maxparts=S;
        maxmod=Q;
    end
end
```

```
%%
%write max modularity matrix to file
maxmodtab=array2table(maxparts);
%set colnames to res
resnames=strcat('res_',strread(num2str(gamma),'%s'));
resnames=strcat(resnames,'_mod_');
resnames=strcat(resnames,strread(num2str(maxmod),'%s'));
resnames=strrep(resnames,'.','_');
maxmodtab.Properties.VariableNames=resnames;

%set rownames to nodes
adjmtable=readtable(inputfile,
'Delimiter','\t','ReadVariableNames',true,'ReadRowNames',true);
maxmodtab.Properties.RowNames=adjmtable.Properties.RowNames;
writetable(maxmodtab,’TwinsUK_Communities_0_4.txt’,'WriteRowNames',true);
exit;
```
