# Supplementary figures and images for: Detection of stable community structures within gut microbiota co-occurrence networks from different human populations
Source: PeerJ. 2018 Feb 7;6:e4303. doi: 10.7717/peerj.4303 (PMC5807925; doi:10.7717/peerj.4303)

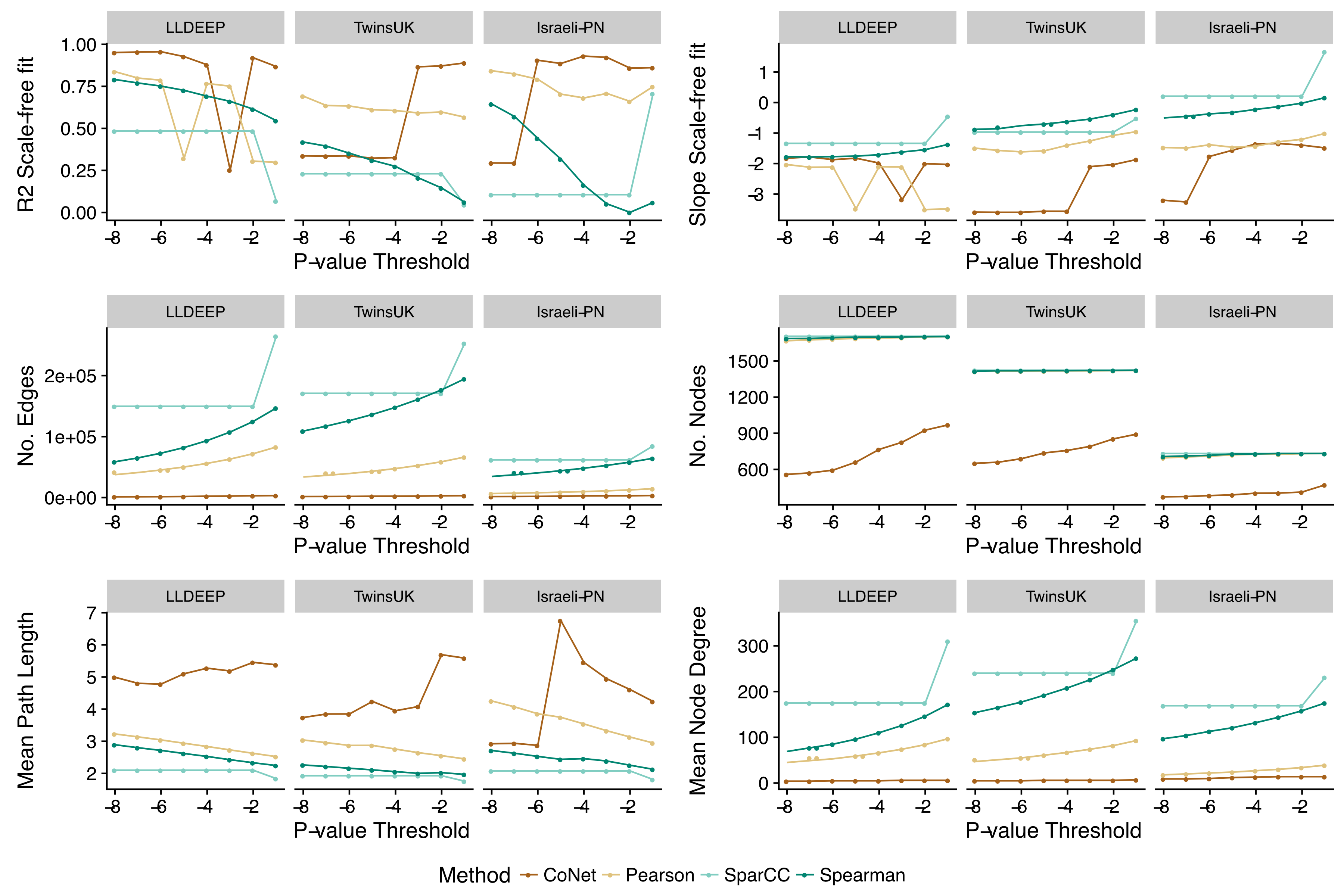

Supplement: Supplemental Information 2 — Model fits and statistics were derived using igraph and WGCNA as for the full intersected networks. Mean path length refers to the mean shortest path length between all pairs of nodes. [file peerj-06-4303-s002.png]

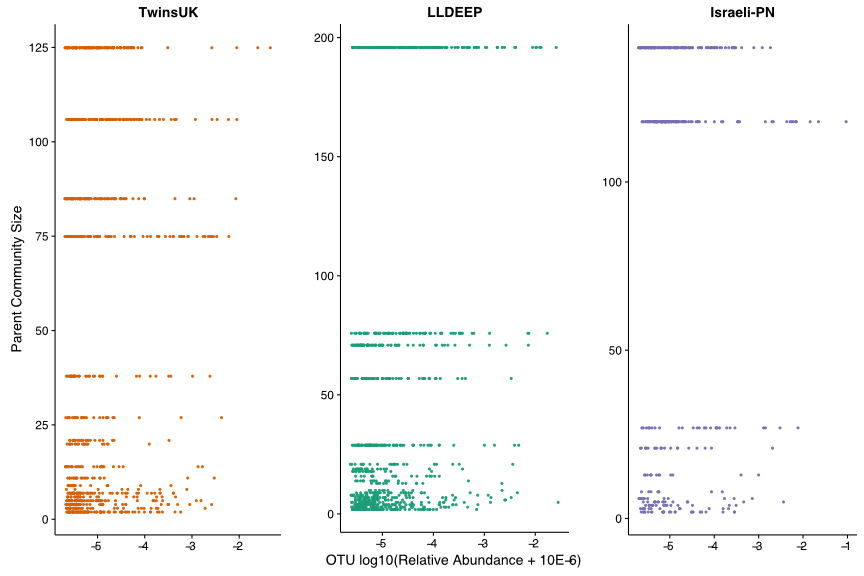

Supplement: Supplemental Information 3 [file peerj-06-4303-s003.png]

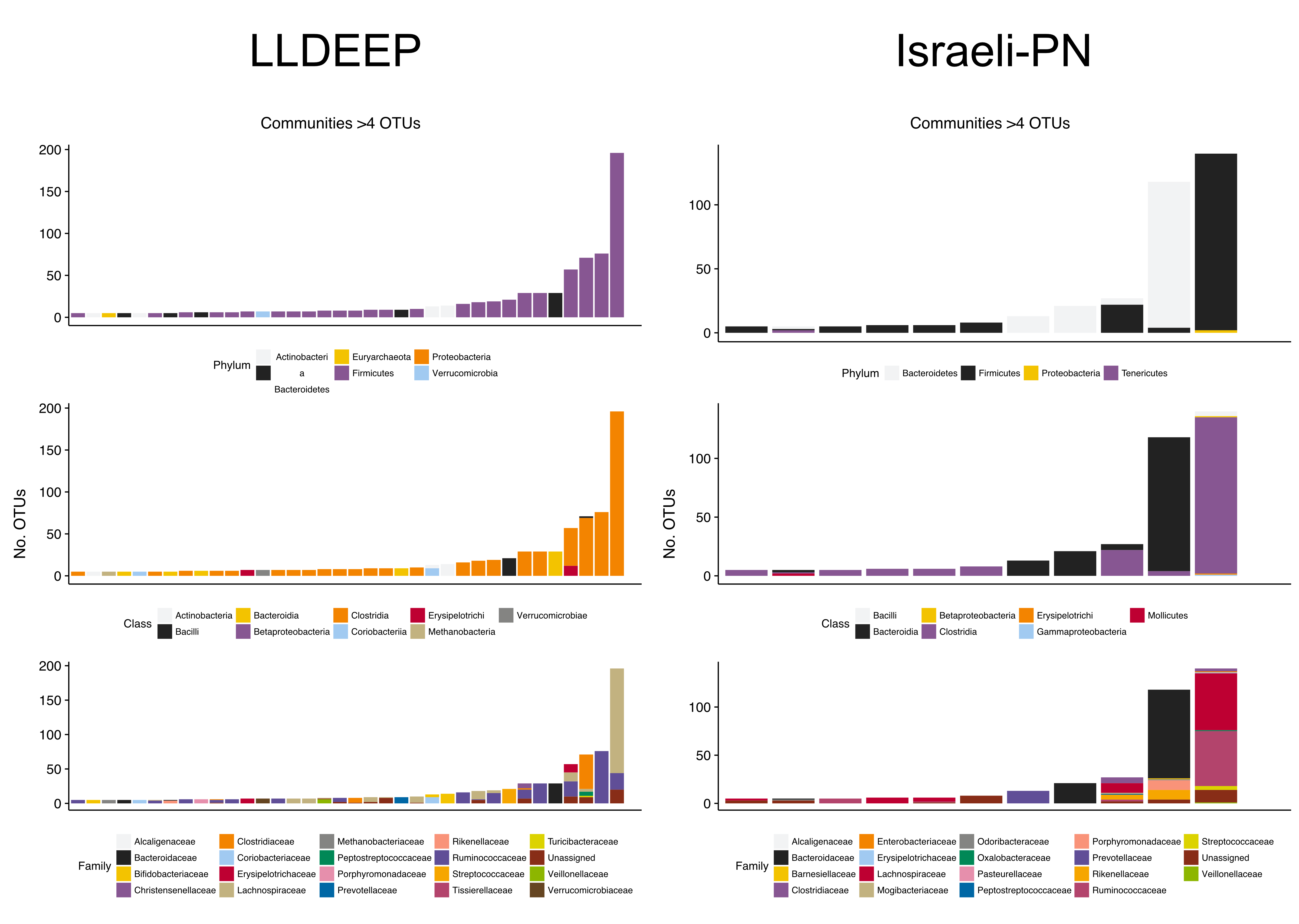

Supplement: Supplemental Information 4 [file peerj-06-4303-s004.png]

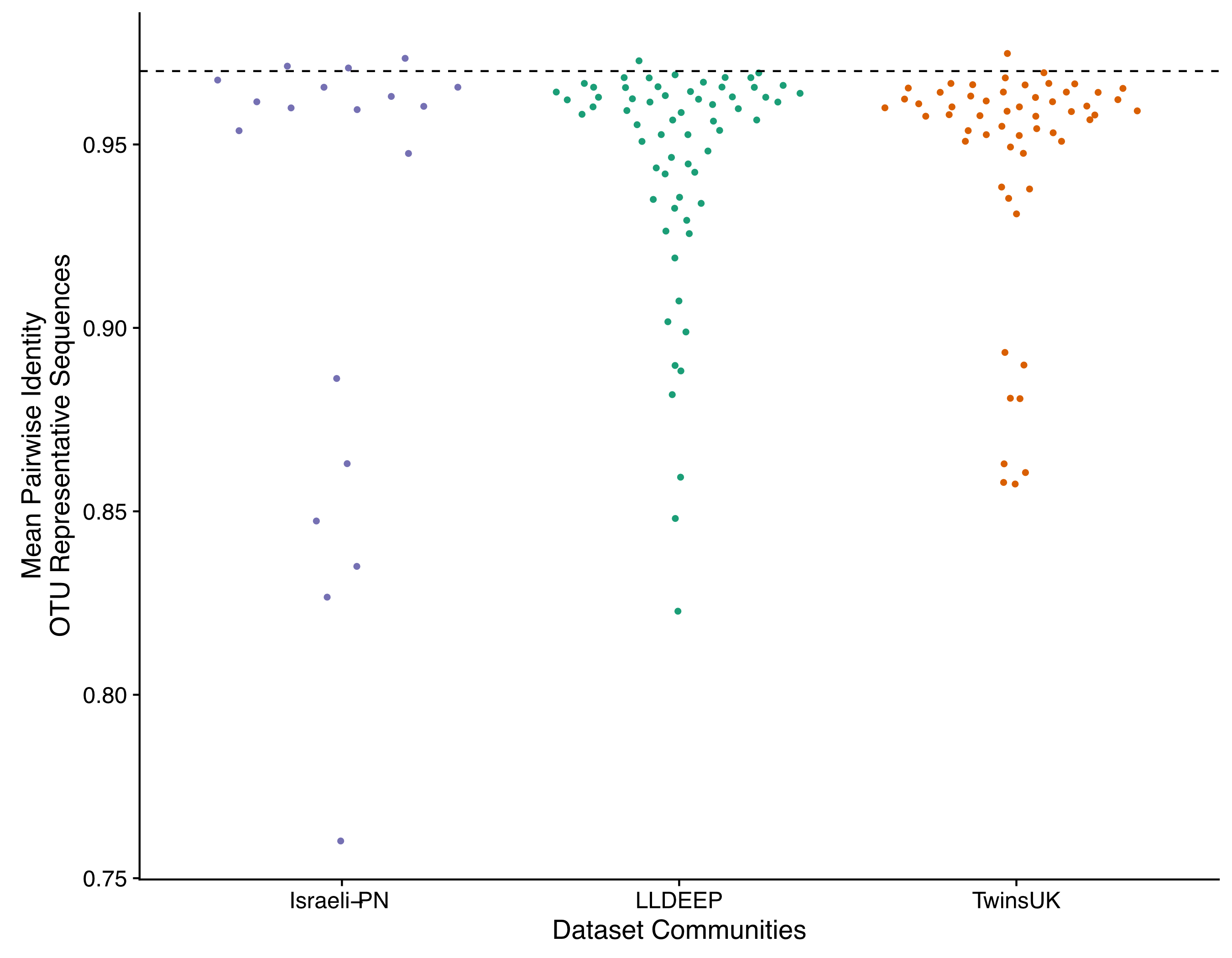

Supplement: Supplemental Information 5 — Shown for all communities with greater than two OTUs. The dashed line represents the 0.97 threshold used to cluster OTUs. [file peerj-06-4303-s005.png]

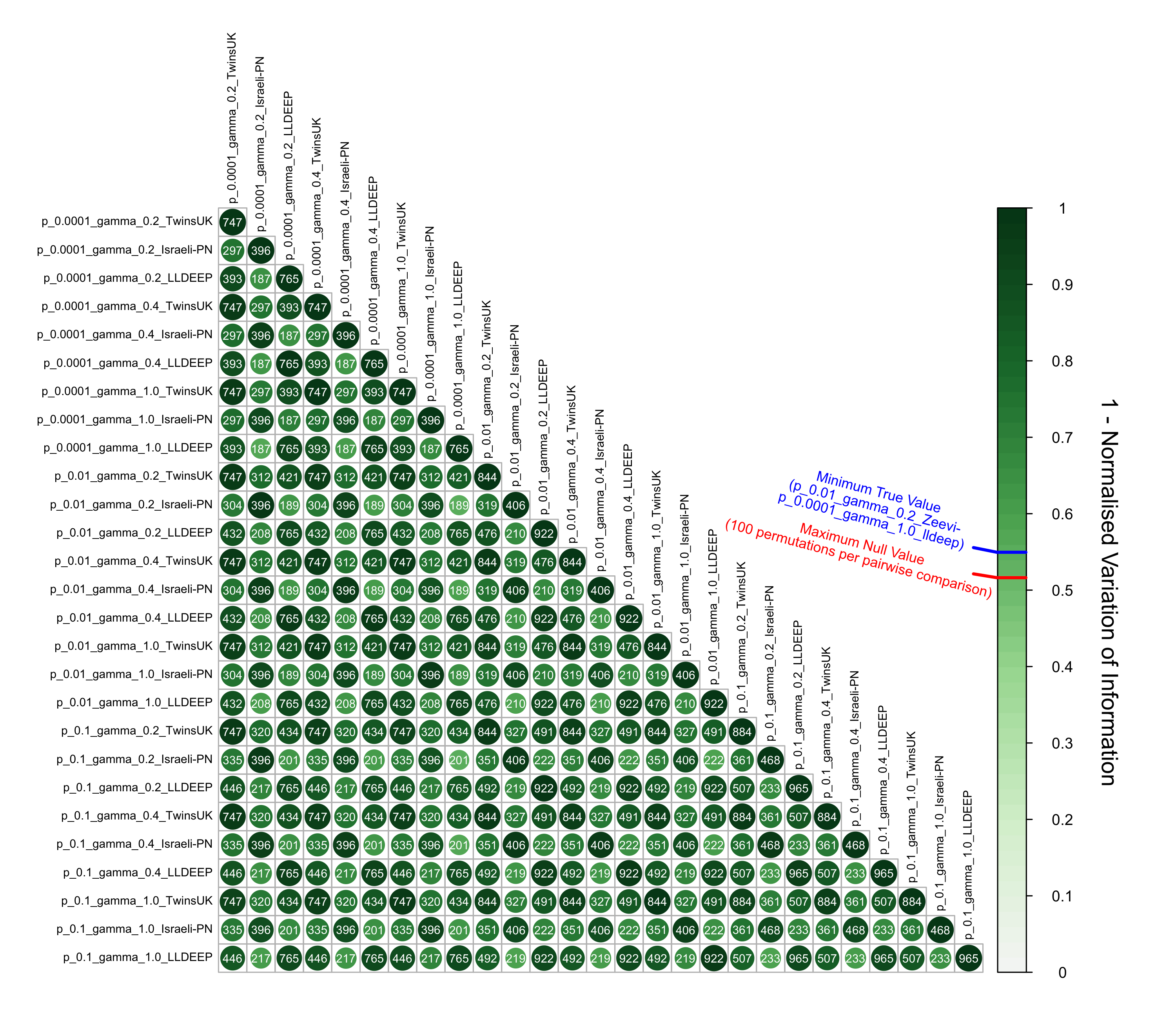

Supplement: Supplemental Information 6 — Variation of information was used to compare each community definition pairwise. For each pairwise comparison the two definitions were shuffled 100 times to generate null estimates. The maximum null estimate observed across all permutations from all comparisons is highlighted on the scale as is the minimum real value observed. Numbers on the points represent the number of overlapping OTUs between the networks used in the comparison. [file peerj-06-4303-s006.png]
